# Retraction Note: Retrospective study of preservation and transection of the round ligament of uterus during laparoscopic transabdominal preperitoneal inguinal hernia repair in adult women

**DOI:** 10.1007/s10029-023-02906-9

**Published:** 2023-10-04

**Authors:** Z. Zhou, C. Tong, L. Tian, X. Zhang, Y. Li, Y. Xiao, L. Yan

**Affiliations:** 1 Department of General Surgery, Shaanxi Provincial People’s Hospital, Xi’an, 710068 China; 2 Yan’an University, Yan’an, China

**Retraction Note: Hernia (2023) 27:1195–1202** 10.1007/s10029-023-02765-4

The authors have retracted this paper because they did not secure ethics approval before conducting post-operative follow-ups with the participants in this study. All authors agree to this retraction.

